# Supplementary material for: US adolescents’ friendship networks and health risk behaviors: a systematic review of studies using social network analysis and Add Health data
Source: PeerJ. 2015 Jun 30;3:e1052. doi: 10.7717/peerj.1052 (PMC4493707; doi:10.7717/peerj.1052)
Supplement: Supplemental Information 1 [file peerj-03-1052-s001.docx]

Appendix A: Sample search strategy (Medline via Ovid)

1. exp sexual behavior/

2. sexuality.mp.

3. sexual.mp.

4. sexual behavior.mp.

5. exp alcohol drinking/

6. drinking.mp.

7. drinking behavior.mp.

8. alcohol.mp.

9. alcohol abuse.mp.

10. alcohol behavior.mp.

11. exp smoke/

12. smoking.mp.

13. smoking behavior.mp.

14. tobacco.mp.

15. tobacco abuse.mp.

16. exp cannabis/

17. marijuana.mp.

18. marijuana abuse.mp.

19. or/1-18

20. exp adolescence/

21. (adolescent$ or teen$ or high school or middle school).ti,ab.

22. (teen$ or youth$).ti,ab.

23. exp friends/

24. (friend$ or friendship$ or (peer adj1 (effect$ or relationship$ or influence$))).ti,ab.

25. ((social adj1 network$) or network analysis$).ti,ab.

26. or/20-25

27. 19 and 26

28. limit 27 to english language

29. limit 28 to yr=“2003-2014”
